# Text Messaging to Motivate Exercise Among Latino Adults at Risk for Vascular Disease: A Pilot Study, 2013

**DOI:** 10.5888/pcd11.140219

**Published:** 2014-10-30

**Authors:** Tracie C. Collins, Frank Dong, Elizabeth Ablah, Deborah Parra-Medina, Paula Cupertino, Nicole Rogers, Carolyn R. Ahlers-Schmidt

**Affiliations:** Author Affiliations: Frank Dong, Elizabeth Ablah, Carolyn R. Ahlers-Schmidt, University of Kansas School of Medicine, Wichita, Kansas; Deborah Parra-Medina, University of Texas Health Science Center, San Antonio Institute for Health Promotion Research, San Antonio, Texas; Paula Cupertino, University of Kansas Medical Center, Kansas City, Kansas; Nicole Rogers, Wichita State University, Wichita, Kansas.

## Abstract

In 2013, we administered a 15-item survey to determine the extent of text message usage among Latino adults in Kansas; for a subset of the survey participants, we also conducted a 6-week pilot trial to determine the effect of text messaging on exercise behaviors. Among the 82 survey participants, 78% had unlimited text messaging. At baseline, all trial participants were at the stage of contemplation; at 6 weeks, one (9%) trial participant remained at the contemplation stage and the other 10 (91%) participants progressed to the action/maintenance/termination stage. Use of text messaging to motivate exercise is feasible and potentially efficacious among Latinos.

## Objective

Peripheral arterial disease (PAD) affects 13.7% of Latino adults (1). An estimated 52% of Latinos are at risk for PAD (2), increasing their risk for amputations (3). Interventions are needed to increase physical activity among Latino adults and reduce the risk for PAD. In the United States, 83% of Latino adults use text messaging compared with 68% of non-Hispanic whites (4). Text messaging has been used to motivate physical activity in other groups (5), but less is known about its impact on Latinos. We sought to determine text messaging’s capacity and its effect on increasing physical activity among Latino adults.

## Methods

We conducted a 2-part study in 2013 that was approved by the Human Subjects Committee at the University of Kansas School of Medicine — Wichita. For recruitment, research assistants traveled to community centers, local businesses, and clinics in Kansas.

For part 1, research assistants administered a 15-item survey in-person or by telephone, in English or Spanish, to determine the rate of cellular telephone and text message usage among Latino adults. The survey was developed by the authors and translated into Spanish by a local consultant. Part 2 was a 6-week trial. Inclusion criterion was being aged 50 years or older with one or more atherosclerotic risk factors (eg, diabetes, smoking, and hypertension). Potential participants were excluded if they had contraindications or were highly motivated to exercise. For this pilot trial, half of survey participants were queried about their interest in physical activity. Reasons for a lack of interest were not obtained. Thirteen were interested and, because of a high motivation to exercise, 2 were ineligible. For the trial, participants completed the Exercise Behaviors Questionnaire (6), which measures minutes per week of exercise, and the Patient-centered Assessment and Counseling for Exercise (PACE) survey (7), which ascertains stage of readiness to engage in physical activity (ie, PACE score). Based on PACE scores, participants were categorized as follows: 1, precontemplation; 2–4, contemplation; 5–8, action/maintenance/termination (ie, highly motivated). The PACE protocol includes scripts designed to encourage exercise and tailored to a PACE score. On the basis of baseline scores, we sent participants English or Spanish text messages with statements pulled directly from the PACE script. Messages were delivered once per day, 5 days per week, for 6 weeks, using the software MessageSpace (8).

Differences between pre- and postexercise behavior scores were calculated for each participant. McNemar tests were used to assess the association between baseline and 6-week PACE scores.

## Results

We recruited 83 participants, and 82 were given the 15-item survey. Mean age of survey participants was 49.2 (standard deviation [SD], 12.0) years, and 70% reported Mexico as their country of origin (Table 1). Among the survey participants, 96% owned a cellular telephone, 89% had texting capacity on their cellphones, and 89% received or sent text messages daily. Furthermore, 81% received at least 1 text message daily, 48% received 5 or more text messages daily, and 78% had unlimited text messaging.

**Table 1:** Use of Cellphones and Text Messaging Among Latino Adults at Risk for Vascular Disease, Kansas 2013

Question	Survey Participants (N = 82)	Trial Participants (N = 11)
**Do you have a cellphone**		
Yes	79	11
No	3	0
**Do you share your cellphone**		
Yes (with someone else living in my home)	14	2
Yes (with a neighbor/friend)	2	0
No	64	8
Unknown	2	1
**For a participant who shares his/her cellphone, number of hours of access to phone each day**		
<1	7	0
1–4	4	0
5–8	2	0
>8	2	2
Missing response	1	0
**Internet on the cellphone**		
Yes	55	6
No	22	3
Unknown	5	2
**Capacity to send text messages**		
Yes	73	10
No	8	1
Unknown	1	0
**Capacity to receive text messages**		
Yes	73	10
No	7	1
Unknown	2	0
**Willing to receive health tips via text messages**		
Yes	65	8
No	3	1
Not sure	9	2
Missing response	5	0
**Type of text message plan**		
Unlimited	64	10
Pay per text	9	0
Not sure	5	0
Missing response	4	1
**Average number of text messages you send and receive each day**		
<1	8	0
1–4	27	4
5 or more	39	5
Unknown	8	2
**Sex**		
Male	24	1
Female	56	10
Unknown	2	0
**Country of origin**		
Mexico	57	8
Honduras	1	0
Peru	4	1
El Salvador	1	0
Cuba	2	0
Columbia	3	1
Paraguay	2	0
Panama	2	0
United States	4	0
Guatemala	1	0
Ecuador	3	1
Unknown	2	0
**Number of years that you have lived in the United States**		
<1	3	0
1–5	2	0
5–10	14	3
>10	61	8
Unknown	2	0
**Primary language spoken at home**		
English	19	1
Spanish	61	10
Unknown	2	0
**Preferred language for receiving text messages**		
English	31	3
Spanish	44	7
Unknown	7	1
Age, mean (SD)	49.2 (12.0) range, 18–72	51.5 (4.1) range, 44–58

Abbreviations: SD, standard deviation.

Of the 82 participants in the survey, 41 were approached to determine their interest in the pilot trial. Thirteen participants were interested and 2 did not meet eligibility because they were highly motivated to exercise. All 11 participants completed the 6-week trial. Mean age of the trial participants was 51.5 (SD, 4.1) years, and 91% of the participants were female. Each of the 11 participants received 30 text messages. Confirming receipt of messages was a feature of MessageSpace. Three participants did not graduate from high school. One participant reported a yearly household income of less than $5,000, 3 reported an income of up to $30,000, and 3 reported an income of $30,000 to $50,000. Two of the participants reported a yearly household income of $50,000 to $100,000. Two participants did not report their income. 

Three participants had one atherosclerotic risk factor and 4 had 3 atherosclerotic risk factors. All 11 participants were at the contemplation stage at baseline. At 6 weeks, one participant remained at the contemplation stage, and the other 10 had moved to the action/maintenance/termination stage. At baseline, the mean minutes per week of exercise was 55.91 (SD, 55.76), which increased to 201.82 (SD, 61.61) minutes per week at 6 weeks (*P* < .003) (Table 2).

**Table 2:** **Six**-**Week Changes in Exercise Behavior and PACE Scores,^a^ 2013**

Outcome Categories	Pre-Exercise	Post-Exercise (6 Weeks)	Difference Between Post and Pre	*P* Value^b^
**Exercise behaviors score, mean (SD)**				
Stretching/strengthening score, minutes/week	19.09 (19.08)	36.82 (59.76)	17.73 (59.17)	.34
Aerobic score, minutes/week	55.91 (55.76)	201.82 (61.61)	145.91 (86.27)	<.001
**PACE stage**				
Contemplation	11 (100%)	1 (9.1%)	NA	NA
Action/maintenance/termination	0	10 (90.9%)	NA	NA

Abbreviations; PACE, Patient-centered Assessment and Counseling for Exercise; SD, standard deviation; NA, not applicable.

a The PACE score had 3 stages: precontemplation, contemplation, and action/maintenance/termination. McNemar test was used to assess the association between baseline and 6-week PACE stage. Due to the zero count in the cross tabulation of PACE stages, no value was produced.

b *P* values from *t *test. The exercise behavior score calculation is based on the Stanford Education Research Center’s scoring method (http://patienteducation.stanford.edu/research/exercise.html). There are 2 types of scores, stretching/strengthening score and aerobic score, each in units of minutes per week.

## Discussion

We found that the capacity for and usage of text messaging among our sample of Latino adults exceeds that of Latino adults nationwide. Furthermore, our pilot demonstrates the feasibility of a scripted counseling approach, delivered using text messaging, to increase physical activity among Latino adults. Scheduled leisure-time physical activity can control atherosclerotic risk factors. Unfortunately, rates of physical activity in the United States are lower for Latinos than for any other racial or ethnic group. According to data from the National Health Interview Survey, nearly 60% of Latinos do not meet federal guidelines for physical activity compared with 47% of non-Hispanic whites (9). Furthermore, Latinos without US citizenship are twice as likely to be inactive as Latinos with US citizenship (10). Evidence that text messaging can promote exercise among older adults from minority races is limited. Buchholz et al (11) conducted a systematic review of physical activity interventions that used text messaging. They reported findings from 10 studies that were largely composed of non-Hispanic whites. In a study of persons with diabetes mellitus, text messaging increased physical activity. In a recent pilot trial, text messaging was successfully used to motivate weight loss (12) among English- or Spanish-speaking participants. The findings from both the systematic review and the recent weight loss pilot trial are similar to ours. However, in contrast to most studies included in the systematic review, we included both English- and Spanish-speaking participants. We also ascertained the rates of cellular telephone and text message usage. Also, in contrast to participants in the weight loss trial, who ranged in age from 21 to 60 years, our participants were aged 50 years or older.

We found that usage of cellular telephones and texting is common among Latino adults. Additionally, text messaging is a viable option to motivate physical activity among Latinos with one or more atherosclerotic risk factors.

## References

[1] Rosero EB , Kane K , Clagett GP , Timaran CH . A systematic review of the limitations and approaches to improve detection and management of peripheral arterial disease in Hispanics. J Vasc Surg 2010;51(4, Suppl):27S–35S. 10.1016/j.jvs.2009.08.085 19939610

[2] Daviglus ML , Talavera GA , Larissa Avilés-Santa M , Allison M , Cai J , Criqui MH , Prevalence of major cardiovascular risk factors and cardiovascular diseases among Hispanic/Latino individuals of diverse backgrounds in the United States. JAMA 2012;308(17):1775–84. 10.1001/jama.2012.14517 23117778 PMC3777250

[3] Rowe VL , Weaver FA , Lane JS , Etzioni DA . Racial and ethnic differences in patterns of treatment for acute peripheral arterial disease in the United States, 1998–2006. J Vasc Surg 2010;51(4, Suppl):21S–6S. 10.1016/j.jvs.2009.09.066 20080006

[4] Pew Research Hispanic Trends Project. Latinos and digital technology; 2010. http://www.pewhispanic.org/2011/02/09/latinos-and-digital-technology-2010/. Accessed May 5, 2012.

[5] Kim BH , Glanz K . Text messaging to motivate walking in older African Americans: a randomized controlled trial. Am J Prev Med 2013;44(1):71–5. 10.1016/j.amepre.2012.09.050 23253653

[6] Lorig K , Stewart A , Ritter P , Gonzalez V , Laurent D , Lynch J . Outcome measures for health education and other health care interventions. Thousand Oaks (CA): Sage Publications; 1996.

[7] Calfas KJ , Long BJ , Sallis JF , Wooten WJ , Pratt M , Patrick K . A controlled trial of physician counseling to promote the adoption of physical activity. Prev Med 1996;25(3):225–33. 10.1006/pmed.1996.0050 8780999

[8] Escobar RD , Akopian D , Parra-Medina D , Esparza L . MessageSpace: a messaging system for health research. Multimedia content and mobile devices, 86670V (March 7, 2013). In: Proceedings SPIE; 8667; 8667 0V. Multimedia content and mobile devices: 4–6 February 2013, Burlingame, California. 10.1117/12.2007530 PMC6859841 31741551

[9] Go AS , Mozaffarian D , Roger VL , Benjamin EJ , Berry JD , Borden WB , Heart disease and stroke statistics — 2013 update: a report from the American Heart Association. Circulation 2013;127(1):e6–e245. Erratum in Circulation 2013;127(1):doi:10.1161/CIR.0b013e31828124ad. Circulation 2013;127(23):e841. 10.1161/CIR.0b013e31828124ad 23239837 PMC5408511

[10] Ahmed NU , Smith GL , Flores AM , Pamies RJ , Mason HR , Woods KF , Racial/ethnic disparity and predictors of leisure-time physical activity among U.S. men. Ethn Dis 2005;15(1):40–52. 15720048

[11] Buchholz SW , Wilbur J , Ingram D , Fogg L . Physical activity text messaging in adults: a systematic review. Worldviews Evid Based Nurs 2013;10(3):163–73. 10.1111/wvn.12002 23746267

[12] Kolodziejczyk JK , Norman GJ , Barrera-Ng A , Dillon L , Marshall S , Arredondo E , Feasibility and effectiveness of an automated bilingual text message intervention for weight loss: pilot study. JMIR Res Protoc 2013;2(2):e48. 10.2196/resprot.2789 24200517 PMC3841356

